# Splitting the Difference: Heterogeneous Soil Moisture Availability Affects Aboveground and Belowground Reserve and Mass Allocation in Trembling Aspen

**DOI:** 10.3389/fpls.2021.654159

**Published:** 2021-05-14

**Authors:** Ashley T. Hart, Morgane Merlin, Erin Wiley, Simon M. Landhäusser

**Affiliations:** ^1^Department of Renewable Resources, University of Alberta, Edmonton, AB, Canada; ^2^Department of Biology, University of Central Arkansas, Conway, AR, United States

**Keywords:** split-pot experiment, drought, structural mass, non-structural carbohydrates, *Populus tremuloides*

## Abstract

When exploring the impact of resource availability on perennial plants, artificial treatments often apply conditions homogeneously across space and time, even though this rarely reflects conditions in natural systems. To investigate the effects of spatially heterogeneous soil moisture on morphological and physiological responses, trembling aspen (*Populus tremuloides*) saplings were used in a split-pot experiment. Following the division of the root systems, saplings were established for a full year and then subjected to either heterogeneous (portion of the root system exposed to non-lethal drought) or homogeneous (whole root system exposed to non-lethal drought or well-watered) treatments. Above- and belowground growth and non-structural carbohydrate (NSC) reserves (soluble sugars and starch) were measured to determine how allocation of reserves and mass between and within organs changed in response to variation in soil moisture availability. In contrast to saplings in the homogeneous drought treatment, which experienced reduced shoot growth, leaf abscission and fine root loss, saplings exposed to the heterogeneous conditions maintained similar aboveground growth and increased root system allocation compared to well-watered saplings. Interestingly under heterogeneous soil moisture conditions, the portion of the root system that was resource limited had no root dieback and increased carbon reserve concentrations, while the portion of the root system that was not resource limited added new roots (30% increase). Overall, saplings subjected to the heterogeneous soil moisture regime over-compensated belowground, both in mass and NSC reserves. These results indicate that the differential allocation of mass or reserves between above- and belowground organs, but also within the root system can occur. While the mechanisms and processes involved in these patterns are not clear, these responses could be interpreted as adaptations and acclimations to preserve the integrity of the entire sapling and suggests that different portions of plant organs might respond autonomously to local conditions. This study provides further appreciation of the complexity of the mechanisms by which plants manage heterogeneous conditions and offers evidence that spatial and temporal variability of resource availability, particularly belowground, needs to be accounted for when extrapolating and modeling stress responses at larger temporal and spatial scales.

## Introduction

As the climate changes, the stochasticity of precipitation events is predicted to increase, and droughts are expected to become more intense and more frequent (IPCC, 2013). These changes have the potential to produce novel soil moisture conditions for many species (Harte and Shaw, 1995; Fridley et al., 2011; Metz and Tielbörger, 2016). Root systems of long-lived plants, such as trees will likely need to acclimate both morphologically and physiologically to these changing soil moisture conditions to ensure long-term survival. Controlled drought studies of potted plants have provided valuable insights into how species may respond to a drier future; however, these studies have several drawbacks. One issue is that the soil medium of pot-grown plants tends to be unrealistically homogenous, yet spatial and temporal heterogeneity in moisture is an inherent characteristic of soil ecosystems (Loranty et al., 2008; Guswa, 2012; Vereecken et al., 2014). Water availability varies both horizontally and vertically throughout a soil profile and is driven by topographical variability (e.g., hillslopes), soil pedogenesis and associated differences in soil properties (Chamran et al., 2002; Tromp-van Meerveld and McDonnell, 2006), vegetation cover and climate dynamics (Berry et al., 2006; Legates et al., 2010; Seneviratne et al., 2010). Therefore, as emphasized by Hutchings and John (2004), controlled studies investigating plant responses to heterogeneous distributions of environmental resources, whether that be moisture, space, light, or nutrients, are necessary to strengthen our understanding of plant growth and behavior, especially for the prediction of species responses and forest dynamics under future climate scenarios.

There has been considerable exploration of how trees and seedlings respond morphologically and physiologically to drought conditions (Breda et al., 2006; Brunner et al., 2015). However, knowledge of plant responses to hetero- vs. homogeneous soil moisture conditions is lacking, in particular how plants may alter the allocation of resources to maintain plant functionality and potentially survival in response to spatial variation. Recognizing how perennial plants balance the allocation of remobilized and newly acquired carbon between and within above- and belowground organs (Bloom et al., 1985; Chapin et al., 1990; Eissenstat, 1997) to structural components (Poorter and Nagel, 2000; Poorter et al., 2012), such as cellulose, hemicellulose and lignin, and/or to non-structural components (Magel et al., 2000; Dietze et al., 2014) such as soluble sugars, starch and secondary compounds, is critical to our understanding of plant stress responses. Based on studies simulating drought conditions that are spatially homogeneous, plants are known to respond to increasing water stress by reducing shoot growth, shedding leaves, reducing stomatal conductance and accumulating solutes in aboveground tissues to maintain turgor and limit xylem cavitation and desiccation (Rood et al., 2000; Arango-Velez et al., 2011; Galvez et al., 2011; Claeys and Inzé, 2013; Buckley et al., 2017). Belowground, as soil water potential decreases and the rhizosphere progressively dries, common responses include structural root growth and/or accumulation of solutes in root tissues to maintain a more negative water potential than the surrounding soil (Meier and Leuschner, 2008; Markesteijn and Poorter, 2009; Galvez et al., 2013). Yet when assessing belowground responses, we must consider that under natural conditions, soil moisture availability is often heterogeneous, and since root systems are capable of exploring and proliferating into favorable patches of soil resources (Drew, 1975; Hutchings and de Kroon, 1994), there is also the potential for distinct morphological and physiological adaptations within a root system depending on the conditions experienced by different parts of a root system (Gersani and Sachs, 1992). Thus, characterizing how carbon is allocated within perennial plants that are subjected to more natural heterogeneous soil moisture conditions could provide more accurate insights into drought avoidance and tolerance mechanisms, as well as how those impact our understanding of hydraulic failure and/or carbon starvation responses (McDowell et al., 2008; Sala et al., 2010; Pinheiro and Chaves, 2011; Adams et al., 2017).

To investigate how newly assimilated and remobilized carbon may be allocated within both aboveground and belowground organs in response to spatially variable soil moisture conditions, we selected a widely distributed tree species, trembling aspen (*Populus tremuloides* Michx.). Trembling aspen is well-known for its clonal root system, which is essential for its regeneration (root suckering) after disturbance (Peterson and Peterson, 1992; DesRochers and Lieffers, 2001; Frey et al., 2004; Wiley et al., 2019). The clonal root system of aspen is large and consists of interconnected lateral roots which can span across large gradients of soil moisture availability (Day, 1944; Snedden, 2013). While responses to both severe and moderate drought have been previously studied in aspen seedlings and large trees (Braatne et al., 1992; Frey et al., 2004; Hogg et al., 2008; Galvez et al., 2011; Anderegg, 2012), no studies have determined how aspen’s drought response—particularly allocation patterns within different portions of a root system—is modulated by the heterogeneity of soil water availability.

The objective of this study was to characterize the morphological and physiological response of aspen saplings that had all or portions of their root systems exposed to progressive, non-lethal drought conditions. Specifically, we assessed the influence of hetero- and homogeneous soil moisture conditions on the aboveground and belowground allocation of non-structural carbohydrate (NSC) components (soluble sugars and starch) and of other mass components (non-NSC, mostly structural) using a split-pot experiment. We hypothesized that saplings subjected to heterogeneous soil moisture conditions would compensate for the partial stress by preferentially increasing carbon (i.e., structural mass and NSCs) allocation toward the root system, accompanied by a decrease in aboveground growth. We also hypothesized that under heterogeneous soil moisture conditions, a sapling would allocate relatively more carbon to the drought exposed portion of the root system compared to a root system that was exposed to a homogeneous drought, as under these soil moisture conditions carbon acquisition and investment into growth would be greatly reduced. Furthermore, we hypothesized that the portion of the root system exposed to non-limiting soil moisture conditions in the heterogeneous treatment would respond similarly to a root system that was exposed to homogeneous non-limiting conditions.

## Materials and Methods

### Split-Pot Design

**FIGURE 1:**
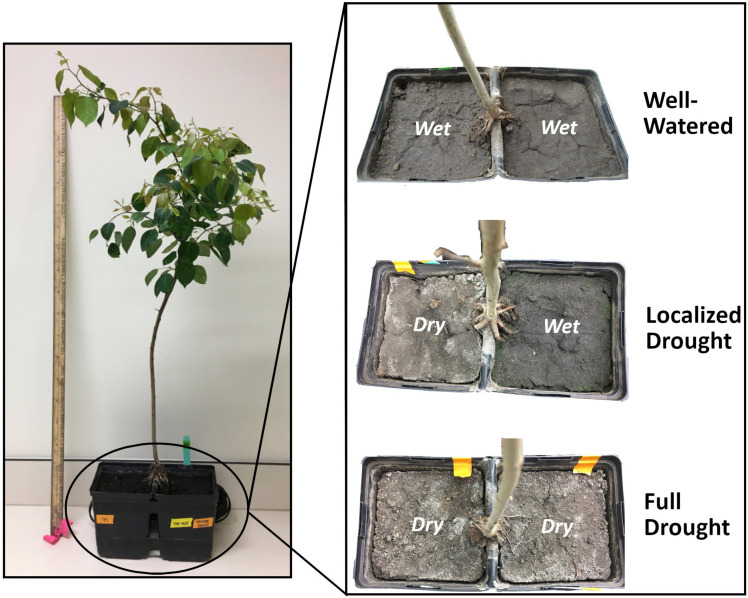
Aspen sapling grown with spatially separated root system using a split-pot design. Aspen saplings were subjected to one of three watering treatments [well-watered (WW), localized drought (LD) or full drought (FD)] characterized by differences in soil moisture availability.

A split-pot apparatus was used to spatially split the root systems of each tree sapling to allow for portions of a common root system to be independently exposed to different soil moisture conditions (Gowing et al., 1990; Fort et al., 1997; Sakuratani and Higuchi, 1999; Hirota et al., 2004; Figure 1). Split-pots were constructed using two square Kordlock pots (10 × 10 × 14 cm tall) stapled together. A square section of rubber liner (Pond Building Series, reinforced PVC pond liner, plant compatible) was glued and sealed with waterproof caulking over the joining portion of the two pots to prevent any water transfer along the edges of the pot. Reinforced tape was wrapped around the two joined pots and a 2.5 cm foam block was glued between the two pots to increase rigidity of the split-pot apparatus. To monitor soil moisture conditions, one half of the split-pot had a matric potential sensor (dielectric water potential sensors MPS2, Decagon Devices, Inc., Pullman, WA, United States) installed through a hole in the pot wall and was sealed into position with waterproof caulking. A piece of very fine mesh was placed at the bottom of each pot to prevent soil loss during watering. A sifted mineral agricultural topsoil with a sandy-loam texture was used as a growing medium. Each pot was then filled with the same weight of sifted soil and compacted to the same soil volume in the pot. A soil water potential response curve was created for the soil at the same bulk density as found in the split-pots, using the pressure extractor method to assess hydraulic properties to ensure a better control of drought conditions (Reynolds and Clarke Topp, 2008; Supplementary Figure 1).

Fifty one-year old nursery grown containerized trembling aspen saplings (6 cm diameter and 15 cm deep) grown from open-pollinated seed sources of Central Alberta (Smoky Lake Forest Nursery, AB, Canada) were used. During planting, the existing root system of each sapling was carefully split by first removing some of the growing medium. Care was then taken to equally divide the root system to accommodate the split-pot design. Separation of the root system was accomplished by dividing the total number of major lateral roots in half and planting them in each pot with the root collar of the sapling sitting on the pot joint (Figure 1). The presence and position of a large root within the split-pot was recorded for each sapling in all treatments to assure similar root distribution across treatments. A small piece of burlap was wrapped around the root collar to cover the exposed section of the root system sitting on the joint between the pots, preventing desiccation and root death during early establishment of the saplings. Saplings were watered regularly and fertilized once using 10-52-10 N-P-K fertilizer (Agrium, Inc., Calgary, AB, Canada). The saplings were kept outside at the University of Alberta (Edmonton, AB, Canada) for 20 weeks (July 4th, 2016–November 21st, 2016) to fully establish and allow the root system to occupy both pots and produce a healthy crown.

To prevent root damage from soil temperatures that were well below freezing (<−5°C), the saplings were moved to a dark growth chamber in November 2016. The chamber was set at a constant temperature of −1°C for a period of 6 weeks to allow saplings to accumulate additional chilling hours. Saplings were watered regularly with a small volume of water during that time, approximately 20 mL weekly, to prevent soil desiccation. After the 6-week dormant period, saplings were exposed to progressively higher air temperatures and increased light conditions over a period of 7 weeks (Supplementary Table 1). In that period, temperature increased to a maximum of 18°C during the day and 16°C at night with 12 h of light [500 μmol m**^–^**^2^ s**^–^**^1^ photosynthetically active radiation (PAR)] which simulated spring conditions (Supplementary Table 1). Relative humidity in the chamber was maintained at 60% throughout the period. During the 7-week spring period, saplings were watered daily, fertilized weekly with 50 mL of 1 g L**^–^**^1^ 15-30-15 N-P-K fertilizer (Agrium, Inc., Calgary, AB, Canada) and pots were rotated weekly to minimize spatial variability. Of the 50 saplings grown initially, only 29 saplings were considered healthy (i.e., successfully flushed and produced new large leaves and elongated new shoots) and were used for the remainder of the experiment.

### Experimental Period and Application of Watering Treatments

The experimental period (4 weeks) started at the beginning of March 2017, during which the growth chamber conditions were set to 20°C both day and night with a 17-h light/7-h dark cycle, a relative humidity at 60% and PAR of 500 μmol m**^–^**^2^ s**^–^**^1^. Initial measurements of height and root collar diameter (RCD) were taken on all saplings generating four groups with similar sapling size distributions (total *n* = 29). Six saplings were harvested at the beginning of the experimental period (Initial). The remaining saplings were separated into treatment groups based on three soil moisture regimes: eight saplings were assigned to have both pots watered to field capacity (homogeneous well-watered treatment: WW); another set of eight saplings had one pot watered to field capacity (wet pot) while the other pot underwent a progressive dry-down (dry pot) (heterogeneous soil moisture: localized drought treatment: LD); and the remaining seven saplings had both pots undergoing a progressive drought (homogeneous full drought treatment: FD) (Figure 1). For all saplings, soil water potential was recorded every 15 min in one of the wet pots in the WW treatment, one of the dry pots in the FD treatment, and in the dry pot of the LD treatment, using the installed soil water potential sensors connected to EM50 dataloggers (Decagon Devices Inc. Pullman, WA, United States). For the first week of the experimental period, saplings received water (only), but then for the remainder of the experiment, water that included a 2 g L**^–^**^1^ solution of 15-30-15 N-P-K fertilizer (Agrium, Inc., Calgary, AB, Canada). Saplings were moved weekly to different positions on the growth chamber benches to reduce any effects of spatial variability in the ambient conditions.

For the WW treatment and the wet pot of the LD treatment, at the start of each daytime period, each pot was watered to saturation and then allowed to drain freely reaching field capacity. For the dry pot of the LD treatment and the FD treatment, a progressive drought was applied. To apply the drought in the LD treatment, the initial starting weight at field capacity for the entire split-pot was determined at the start of the experimental period. During the experimental period, the wet portion of the split-pot was always re-watered to field capacity (watered to saturation and allowed to completely drain) at the beginning of the day, then the entire split-pot was weighed, and the difference to the initial (previous) weight was attributed to the water loss from the dry pot only. The dry pot was then watered with half of the water loss amount, based on the weight lost, thus contributing to a gradual decrease in soil water potential over a period of 4 weeks (Supplementary Figure 2). For the last 2 weeks of the experimental period, the soil water potential in the dry pots was maintained between −700 to −1200 kPa, with the pots receiving only small water additions (<5 g) during the last 4 days (Supplementary Figure 2). Soil water potentials were maintained within this range to avoid catastrophic drought-induced cavitation, as previous research has demonstrated that an average xylem pressure below −1200 kPa can produce a loss of hydraulic conductivity greater than 50% in aspen (Plavcova and Hacke, 2012; Fichot et al., 2015; Schreiber et al., 2016). A similar watering strategy was applied to the FD treatment; however, here both sides of the split-pot were subjected to the same progressive drought, with both pots receiving water in the amount replacing only half the water lost from the previous day and then maintained at the same range of water potential to achieve a similar drought intensity at the pot-level compared to the LD dry pot (Supplementary Figure 2).

### Measurements

To monitor physiological responses, net assimilation and stomatal conductance were measured with a LI-6400XT portable photosynthesis system (LiCor, Inc., Lincoln, NE, United States) once a week throughout the experimental period on three saplings (two leaves each) randomly selected within each treatment. Leaf chamber light was set at 800 μmol m**^–^**^2^ s**^–^**^1^, CO_2_ was set at 400 ppm, incoming relative humidity at 60% and leaf temperature at 20°C to mimic the conditions in the growth chamber. Net assimilation and stomatal conductance were measured 2 h after the beginning of the daytime period.

At the end of the experimental period, the final height and RCD were measured on all saplings at harvest. Saplings were separated into leaves, stem (old and new primary growth) and the two portions of the split root system. Projected leaf area was measured for each sapling in the LD and the WW treatments using a LI-3100 area meter (LiCor, Inc., Lincoln, NE, United States). Leaf area could not be measured for the FD saplings, as dead leaves in the FD treatment were too brittle to measure. The portions of the root system were extracted separately from each side of the split-plot and kept separated. After careful removal of all soil, fresh root volume, which more closely represents the root surface area (estimate of water uptake potential), was measured for each portion of the split root system via the water displacement method (Harrington et al., 1994). All collected tissues from each sapling were dried for 1 h at 100°C to denature enzymes, followed by 48 h at 70°C to constant weight. Dried root samples were separated into coarse roots (diameter > 1 mm) and fine roots (diameter < 1 mm). All dried material was weighed, and samples were ground to a 40-mesh (0.4 mm) using a Thomas mini Wiley mill (Thomas Scientific, Inc., Swedesboro, NJ, United States) for subsequent NSC analysis.

Non-structural carbohydrates (NSC) were analyzed following the protocol described in Landhäusser et al. (2018). In brief, total soluble sugars were extracted in 80% hot ethanol followed by a phenol-sulfuric assay to determine their concentration colorimetrically by measuring the absorbance at 490 nm with a spectrophotometer. To determine starch concentration, the remaining pellet was digested with α-amylase (Sigma cat. no. A4551) and amyloglucosidase (Sigma cat. no. ROAMYGL). A peroxide-glucose oxidase/o-dianisidine reagent was then added to the resulting glucose hydrolysate. After incubation, concentrated sulfuric acid was added before measuring absorbance at 525 nm. Absorbance values were used to calculate sugar and starch concentrations by comparison with standard curves and expressed as percent of sample dry weight.

### Calculations and Statistical Analyses

The following calculations were used to compare the effects of heterogeneous soil moisture vs. uniform soil moisture on the allocation of structural and non-structural (soluble sugars and starch) components between aboveground and belowground tissues and within the root system (i.e., between the split-pots). Treatment effects on growth were assessed using sapling height, RCD, and biomass. Height and RCD growth during the experimental period were calculated by subtracting the initial height and RCD measured on each treated sapling at the beginning of the experiment from the final height and RCD of that same sapling. To evaluate changes in leaf and stem mass that occurred during the 4-week experimental period (i.e., new leaf and new stem growth), the average of initial measurements, taken from the six destructively sampled saplings at the beginning of the experimental period, were subtracted from the individual final treatment measurements of leaf mass (which included dead leaves in the FD treatment) and the mass of primary stem growth. Specific leaf area was calculated by dividing total leaf area by total leaf dry mass. To determine mass allocation in saplings, the ratio of leaf, stem or root mass to total sapling mass was calculated for each sapling. Leaf, stem and root NSC mass (pools) were estimated by multiplying the total sugar and starch concentrations by the total dry mass of each sampled organ. Further, the remaining mass (hereafter called structural mass) that was not related to reserve mass of each organ was estimated by subtracting the respective NSC mass from the total dry mass of each organ. These measures were estimated to evaluate any differences in leaf structural mass, stem structural mass and root structural mass in response to our treatments. In addition, root structural density was calculated as a ratio of the structural mass of the entire root system (fine and coarse roots combined) over the measured root volume (fine and coarse roots combined), to explore potential changes in the morphological composition of the root system.

All data were analyzed using R statistical software v3.5.1 (R Development Core Team, 2018). Assumptions of normality and homoscedasticity were tested using the Shapiro-Wilks test and Levene’s test for parametric analyses. If these assumptions were not met, removal of outliers and transformations were applied. The soil water potential data was fit with a logistic non-linear model using the nlme package (Pinheiro et al., 2016) to show the gradual dry down of soil within the split-pots (Supplementary Figure 2). No differences in root measures between the two sides of the split-pots were found in either well-watered or full-drought treatments using *t*-tests (data not shown; *p* > 0.1). Thus, in subsequent analyses, the two sides of the split-pots in these treatments are considered equivalent. Two statistical analyses were applied to the data. First, to test for differences among the initial harvest and three treatments for aboveground and combined belowground measures, one-way ANOVA was used followed by pairwise *post-hoc* tests with a Benjamini-Hochberg adjustment using the emmeans package (Lenth, 2019). Second, to understand how the localized drought (LD) treatment impacted *within*-root system response, a linear mixed model was used with pot-type (LD-dry, LD-wet, FD, WW) as a fixed factor. Individual sapling was included as a random factor to account for the fact that the same individual was repeatedly measured (2 pots per sapling). The initial presence/absence of a large root within a pot was also included as a random factor to account for the fact that a large root could impact pot-level variables like final root mass, volume, etc. The post-hoc tests for these models were restricted to the following planned comparisons: (1) dry pots vs. the wet pots of the LD treatment, (2) dry pots of the LD treatment vs. the FD treatment pots, and (3) wet pots of the LD treatment compared to the WW treatment pots. The Benjamini-Hochberg adjustment was used with the emmeans package. Differences among treatments or between sides of the split-pot were considered statistically significant at α = 0.1. Estimated marginal means and standard errors are reported in the Results and Discussion sections.

## Results

### Growth and Mass Allocation

Overall, saplings exposed to the localized drought (LD) had various aboveground measures that were similar in comparison to the well-watered (WW) saplings but were greater than those measures in saplings of the full drought (FD) treatment. Although average height growth did not differ among the three treatments (*p* > 0.18; Table 1), the LD and the WW saplings had overall larger RCDs, with over three times more RCD growth compared to the FD saplings (*p* < 0.01; Table 1). Total aboveground dry mass was approximately 30% greater in the LD and the WW treatments compared to the FD treatment, for which no significant increase in aboveground mass occurred (*p* < 0.01; Table 1). Saplings in the LD and the WW treatments produced 1.74 and 2.34 g, respectively, of new leaf mass during the 4-week experimental period, while saplings in the FD treatment did not produce any new leaves (*p* = 0.03; Table 1). Saplings in the FD treatment also experienced partial browning of pre-existing leaves and significant leaf abscission prior to harvest. Specific leaf area was similar between the LD and the WW saplings (Table 1). During the experimental period, stem growth of the WW saplings was 1.27 g, which was similar to the stem growth of the LD saplings (0.87 g) and greater than the stem growth of the FD saplings (0.36 g; *p* < 0.01) (Table 1). The allocation of mass to leaves (25%) and stems (30%) was statistically similar among the three soil moisture treatments (Table 1), although the allocation to leaves in the FD treatment is likely lower if only live leaf mass had been considered. During the period when the soil water potential was maintained between −700 to −1200 kPa, there were no differences in the net assimilation rate and stomatal conductance between the LD saplings (7.74 ± 1.23 μmol_CO__2_ m**^–^**^2^ s**^–^**^1^ and 0.196 ± 0.029 mmol_H__2O_ m**^–^**^2^ s**^–^**^1^, respectively) and the WW saplings (8.62 ± 1.23 μmol_CO__2_ m**^–^**^2^ s**^–^**^1^ and 0.243 ± 0.029 mmol_H__2O_ m**^–^**^2^ s**^–^**^1^, respectively), whereas the FD saplings had significantly lower net assimilation and stomatal conductance compared to the other two treatments (−1.50 ± 1.73 μmol_CO__2_ m**^–^**^2^ s**^–^**^1^ and 0.019 ± 0.041 mmol_H__2O_ m**^–^**^2^ s**^–^**^1^, respectively, both *p* < 0.01).

**TABLE 1:** Estimated marginal means (±1 standard error) of aboveground and belowground variables: height growth (cm), root collar diameter (RCD) growth (mm), total sapling mass (g), total aboveground mass (g), new stem growth (g), leaf mass (g), new leaf growth (g), specific leaf area (cm^2^ g^–1^), total root mass (g), total coarse root mass (g), total fine root mass (g), total root volume (cm^3^), structural root density (g cm^–3^), leaf mass ratio (%), stem mass ratio (%) and root mass ratio (%) prior to (INITIAL) and after three watering treatments (FD, full drought; LD, localized drought; WW, well-watered).

	INITIAL	FD	LD	WW
Height growth (cm)	NA	3.3(1.3)a	4.1(1.5)a	8.8(2.6)a
RCD growth (mm)	NA	0.72(0.26)b	2.50(0.24)a	2.62(0.24)a
Total sapling mass (g)	21.8(2.1)b	22.6(2.0)b	33.5(1.9)a	32.1(1.9)a
Total aboveground mass (g)	12.2(1.2)b	12.5(1.1)b	18.4(1.1)a	19.7(1.1)a
New stem growth (g)	NA	0.36(0.20)b	0.87(0.18)ab	1.27(0.18)a
Leaf mass (g)	6.29(0.58)b	5.40(0.56)b	8.03(0.53)a	8.63(0.53)a
New leaf growth (g)	NA	0.27(0.46)b	1.74(0.43)a	2.34(0.43)a
Specific leaf area (cm^2^ g**^–^**^1^)	162.9(6.4)a	NA	125.5(4.7)b	123.7(4.7)b
Total root mass (g)	9.58(1.25)b	10.13(1.16)b	15.03(1.08)a	12.37(1.08)ab
Total coarse root mass (g)	3.94(0.65)*c*	5.66(0.61)*bc*	8.03(0.57)a	7.04(0.57)ab
Total fine root mass (g)	5.64(0.67)ab	4.47(0.62)b	7.00(0.58)a	5.33(0.58)ab
Total root volume (cm^3^)	59.1(6.1)a	37.2(5.7)b	63.1(5.3)a	58.3(5.3)a
Structural root density (g cm**^–^**^3^)	0.141(0.01)*c*	0.247(0.01)a	0.203(0.009)b	0.186(0.009)b
Leaf mass ratio (%)	29(2)a	24(2)a	24(2)a	28(2)a
Stem mass ratio (%)	26(2)b	31(2)a	31(2)a	34(2)a
Root mass ratio (%)	44(2)ab	45(2)a	45(2)a	38(2)b

*Letters represent statistical difference (n = 29; α = 0.1) among treatments using post-hoc comparisons with a Benjamini-Hochberg adjustment. NA, not applicable.*

Localized drought saplings had a total root dry mass (both pots combined) of 15 g, similar to the 12.4 g of WW saplings, and nearly 50% more compared to the FD saplings (10.1 g, *p* = 0.01) (Table 1). Full drought saplings had a total root dry mass that was similar to the initial saplings (Table 1). However, when comparing total root volume which relates to root surface area and its potential for water uptake, FD saplings also had a total root volume of 37.2 cm^3^ at harvest, which was significantly lower than the LD and the WW saplings (63.1 cm^3^ and 58.3 cm^3^, respectively, *p* = 0.01 and *p* = 0.02, respectively), but also significantly lower than the average initial root volume of 59.1 cm^3^ (*p* = 0.03) (Table 1). Root structural density of the root system, a measure that indicates potential changes in root system morphology, was overall higher in all three experimental treatments after the 4-week experimental period compared to the start of the study (0.141 g cm**^–^**^3^; *p* < 0.01). However, while the root systems of the LD and WW saplings had similar root structural density at the end of the experiment (0.203 and 0.186 g cm**^–^**^3^, respectively), root structural density in FD saplings was higher than both treatments (0.247 g/cm^3^; both *p* < 0.01) (Table 1). Overall, the LD saplings and the FD saplings allocated a greater amount of mass toward the root system (45%) compared to the WW saplings (38%) (*p* = 0.07; Table 1). This suggests that the WW saplings allocated more mass to the aboveground variables, such as height growth, stem and leaf mass which all tended to be greater in the WW saplings, but we could not detect significant statistical differences between the LD and the WW saplings (*p* = 0.2, *p* = 0.1, and *p* = 0.1, respectively).

**FIGURE 2:**
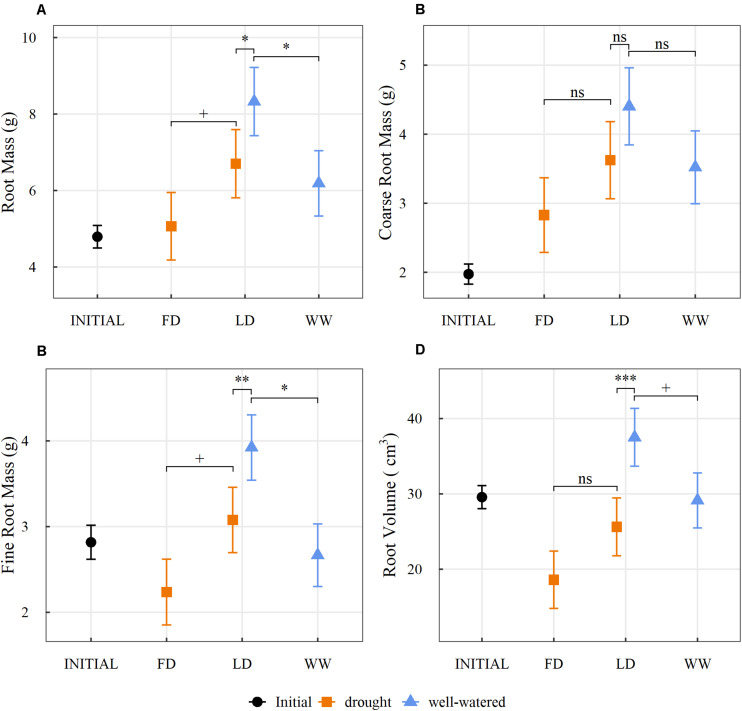
Estimated marginal means (±1 standard error) of **(A)** root mass (g), **(B)** coarse root mass (g), **(C)** fine root mass (g) and **(D)** root volume (cm^3^) within the split root system of saplings, prior to (INITIAL) and after the three watering treatments (FD, full drought**;** LD, localized drought**;** WW, well-watered) (orange: dry, blue: well-watered). Statistical differences between the three planned comparisons (FD vs. LD-dry, LD-dry vs. LD-wet, WW vs LD-wet) are indicated by + for *p* < 0.1, * for *p* < 0.05, ** for *p* < 0.01, *** for *p* < 0.001 and ns for no significance (*n* = 29; α = 0.1).

A closer analysis of belowground measurements between the dry and the wet root system portions in the split-pots of the LD saplings revealed distinct patterns of allocation. Under the localized drought conditions, saplings allocated more mass to the roots within the wet pot (8.33 g) than to the roots within the dry pot (6.70 g; *p* = 0.01; Figure 2A). This greater root mass in the wet pot can be attributed to an increase in fine root production compared to the dry pot (*p* < 0.01; Figure 2C). The portion of the root system contained in the wet pot of the LD treatment also had a greater mass (8.33 g), comprised of significantly more fine roots (*p* = 0.02; Figure 2C) than either section of the root systems in the WW treatment (6.19 g; *p* = 0.03) (Figure 2A). The root system portion contained in the dry pot of the LD treatment was greater in mass, with a significant increase in fine roots (*p* = 0.09; Figure 2C), compared to either root system portion in the FD treatment (5.06 g; *p* = 0.08; Figure 2A). When comparing root system volumes, which relate to root surface area and potential for water uptake, across the split-pots, the root system portion in the wet pot of the LD treatment had a greater volume (37.51 cm^3^) than the root portion in the dry pot (25.63 cm^3^; *p* < 0.01; Figure 2D). However, the root volume in the wet pot of the LD treatment was greater than the volume of the root portions measured in the WW treatment (29.14 cm^3^; *p* = 0.08; Figure 2D). Although there was a trend for the dry portion of the root system in the LD treatment to have a greater volume (25.63 cm^3^) than in the FD treatment (18.6 cm^3^) the difference was not statistically significant (*p* = 0.12; Figure 2D).

### Non-structural Carbohydrate Concentrations

At the end of the 4-week period, there were only a few differences in the starch concentrations in the aboveground tissues among the three watering treatments, while stark differences existed in the root NSC concentrations in response to the different soil moisture conditions. Aboveground, there were no differences in total NSC (sum of starch and soluble sugars) and sugar concentrations in leaf tissue among the three watering treatments, and these concentrations did not differ from the initial measurement (Table 2). However, the FD saplings had lower leaf starch concentrations (0.20%) compared to the LD and the WW saplings (0.78 and 0.66%, respectively, *p* < 0.01 and *p* = 0.01, respectively, Table 2). In comparison to the initial measurement, stem NSC concentrations did not change in LD saplings (10.28% vs. 9.66%, respectively), WW saplings (8.27%) or FD saplings (8.52%) (Table 2). Among treatments, the LD saplings had a similar stem starch concentration (1.31%) compared to WW saplings (0.73%; *p* = 0.12), yet a significantly greater starch concentration compared to the FD saplings (0.05%; *p* < 0.01) (Table 2). Only the LD saplings increased stem starch concentrations over the initial measurement of 0.38% (*p* = 0.01; Table 2). Stem sugar concentrations prior to the start of the experiment were 9.87% which decreased slightly during the experimental period (*p* < 0.06), but no differences were detected in soluble sugar concentrations in the stems among the three watering treatments (Table 2).

**TABLE 2:** Estimated marginal means (±1 standard error) of starch and soluble sugar concentration (% dry weight) for leaf and stem tissue, and of total non-structural carbohydrate (NSC) concentration (% dry weight) of leaf, stem and root (both pots combined) tissue, prior to (INITIAL) and after the three watering treatments (FD, full drought; LD, localized drought; WW, well-watered).

Organ		INITIAL	FD	LD	WW
Leaf	Starch	0.75(0.15)a	0.20(0.10)b	0.78(0.13)a	0.66(0.12)a
	Sugar	13.78(0.58)a	13.53(0.54)a	12.56(0.50)a	12.69(0.50)a
	NSC	14.62(0.64)a	13.75(0.63)a	13.37(0.60)a	13.36(0.60)a
Stem	Starch	0.38(0.18)b	0.05(0.12)b	1.31(0.26)a	0.73(0.19)ab
	Sugar	9.87(0.55)a	8.29(0.43)b	8.19(0.39)b	7.34(0.35)b
	NSC	10.28(0.61)a	8.52(0.62)a	9.66(0.58)a	8.27(0.58)a
Root	NSC	12.95(1.64)a	12.85(1.58)a	15.49(1.48)a	12.90(1.48)a

*Letters represent statistical difference (n = 29; α = 0.1) among treatments using post-hoc comparisons with a Benjamini-Hochberg adjustment.*

**FIGURE 3:**
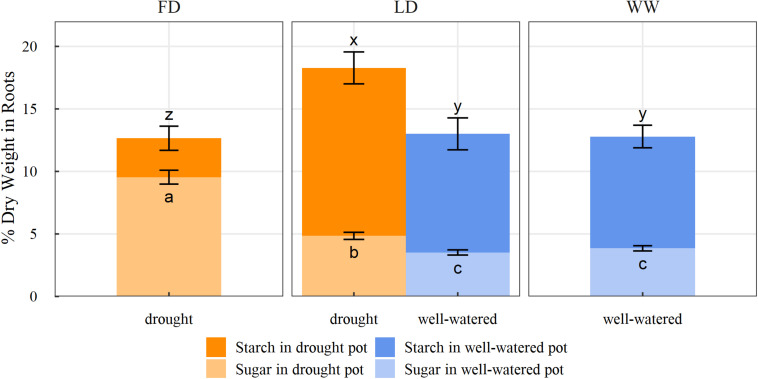
Estimated marginal means (±1 standard error) of root starch and soluble sugar concentration (% of dry weight) within the split root systems of saplings subjected to three watering treatments (FD: full drought, LD: localized drought, WW: well-watered), (light orange: sugar concentration of roots in drought pot, dark orange: starch concentration of roots in drought pot, light blue: sugar concentration of roots in well-watered pot, dark blue: starch concentration of roots in well-watered pot). Letters indicate statistical differences among treatments and pot watering regimes (*n* = 29; α = 0.1) using pairwise comparisons with a Benjamini-Hochberg adjustment.

Belowground, the total NSC concentrations found across the entire root system (both pots combined) did not differ from the initial measurements or among the three watering treatments (Table 2). However, when the NSC concentrations of the root systems were compared between the split-pots, the roots in the dry pot of the LD treatment had higher NSC concentrations (18.35%) than the roots in the wet pot of the LD treatment (13.03%; *p* < 0.01; Figure 3). Furthermore, the roots in the dry pot of the LD treatment had higher NSC concentrations than the roots in the FD treatment (12.82%; *p* = 0.03) (Figure 3). In contrast, NSC concentrations of the roots in the wet pot of the LD treatment (13.03%) did not differ from those in the WW treatment (12.83%) (Figure 3). When broken down into soluble sugar and starch, roots in the dry pot of the LD treatment had higher soluble sugar (4.85%) and starch (13.44%) concentrations than the roots in the wet pot, with 3.51 and 9.49%, respectively (*p* < 0.01 and *p* = 0.05, respectively; Figure 3). The roots in the wet pot of the LD treatment had soluble sugar and starch concentrations similar to the roots in the WW treatment. The roots in the FD treatment had higher soluble sugar (9.54%) but lower starch (3.11%) concentrations compared to the roots in the dry pot of the LD treatment (both *p* < 0.01; Figure 3).

### Allocation to Structural Mass and Non-structural Carbohydrate Pools

The NSC concentrations in the leaves, stem and roots of each individual sapling were used to estimate the structural mass and the NSC pool sizes and their relative allocation (% of total) in response to the soil moisture treatments. Of the total structural pool, the LD saplings allocated 24.2% to leaves, 32.1% to the stem, and 43.7% to roots (Table 3). There were no differences in the relative allocation of structural mass to leaves among the three soil moisture treatments (Table 3). Well-watered saplings allocated slightly more structural mass to the stem in comparison to the LD saplings (*p* = 0.09; Table 3). However, while the relative allocation of structural mass to the root system was similar for the LD and the FD saplings, the WW saplings allocated less structural mass to the root system (37.2%) compared to both treatments (both *p* = 0.03; Table 3). Of the total NSC pool (sum of soluble sugars and starch), the LD saplings allocated 25.3% to leaves, 22.3% to the stem, and 52.4% to roots (Table 3). The relative allocation of NSC to leaves, stems and the root systems were similar among the three soil moisture treatments (Table 3).

**TABLE 3:** Estimated marginal means (±1 standard error) of the relative allocation (% of total sapling pool type) of structural and non-structural carbohydrate (NSC) pools for leaves, stem and roots prior to (INITIAL) and after the three watering regimes (FD, full drought; LD, localized drought; WW, well-watered).

Organ	Pool type (%)	INITIAL	FD	LD	WW
Leaf	Structural	28.4(1.7)a	23.1(1.7)a	24.2(1.6)a	26.9(1.6)a
	NSC	33.8(3.7)a	29.3(3.7)a	25.3(3.5)a	33.2(3.5)a
Stem	Structural	27.5(1.4)*c*	32.9(1.2)ab	32.1(1.1)b	35.7(1.2)a
	NSC	21.2(3.6)a	21.6(3.3)a	22.3(3.1)a	24.5(3.1)a
Root	Structural	44.1(2.0)a	44.0(2.0)a	43.7(1.8)a	37.2(1.8)b
	NSC	44.3(4.4)a	47.6(4.5)a	52.4(4.2)a	41.4(4.2)a

*Letters represent statistical difference (n = 29; α = 0.1) among treatments using post-hoc comparisons with a Benjamini-Hochberg adjustment.*

**FIGURE 4:**
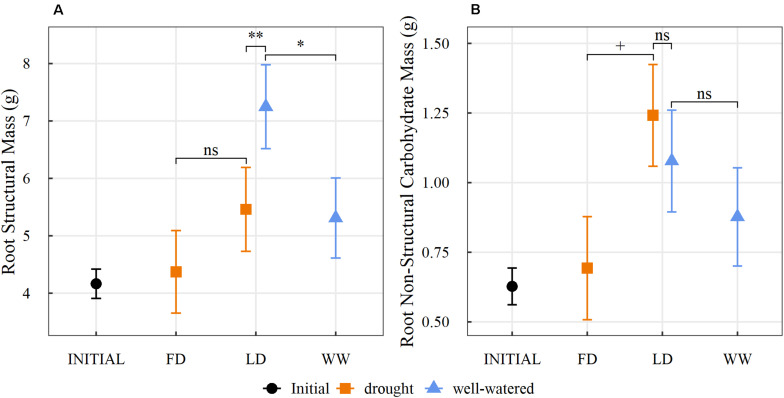
Estimated marginal mean (±1 standard error) of **(A)** root structural mass (g) (the non-reserve portion of dry root mass) and **(B)** root non-structural carbohydrate mass (g) within the split root system for saplings prior to (INITIAL) and after the three watering treatments (FD: full drought, LD: localized drought, WW: well-watered) (orange: dry, blue: well-watered). Statistical differences between the three planned comparisons (FD vs. LD-dry, LD-dry vs. LD-wet, WW vs. LD-wet) are indicated by + for *p* < 0.1, * for *p* < 0.05, ** for *p* < 0.01 and ns for no significance (*n* = 29; α = 0.1).

Differences in the structural mass within the two portions of the split root system in the LD saplings were driven by the localized soil moisture conditions. Roots in the dry pot had less structural mass than the roots contained within the wet pot (*p* < 0.01) but did not differ from the amount of structural mass of a root system portion found in the FD treatment (Figure 4A). In contrast, the roots in the wet pot of the LD treatment had 30% more structural mass compared to the roots in the WW treatment (*p* = 0.01; Figure 4A). LD saplings had similar NSC mass in their dry and wet pots, however, the dry portion of the root system had over double the NSC mass compared to either half of the root systems of the FD saplings (*p* = 0.06; Figure 4B). The NSC mass of roots in the wet pot of the LD treatment was not significantly different from either half of the root system in the WW treatment (*p* = 0.4; Figure 4B).

## Discussion

Our study demonstrates that the root systems of aspen saplings subjected to heterogeneous water availability (LD treatment) responded unlike saplings with root systems that were exposed to homogeneous soil moisture conditions [full drought (FD) or well-watered (WW)]. Based on the heterogeneous conditions in the LD treatment, saplings responded quickly by partitioning structural mass and NSC functionally across and within organs. As expected, saplings exposed to either the full or the localized water limitation increased overall allocation toward the root system (45%) compared to saplings growing in non-limiting conditions (38%). However, the saplings exposed to the full drought experienced reduced gas exchange, terminated aboveground growth, shed leaves and lost root volume. In contrast, the saplings exposed to the LD treatment maintained gas exchange and aboveground growth similar to the WW saplings, avoided root loss in the dry soil, while increasing root structural mass and volume in the wet soil. The different responses of the roots in the dry vs. wet soil under the localized drought treatment suggests the potential for some autonomy within root systems to adaptively adjust allocation depending on the soil conditions individual roots are exposed to.

While the responses of aspen exposed to either homogeneous or heterogeneous drought appear to be consistent with the concept of functional equilibrium of biomass allocation or optimal partitioning theory (Brouwer, 1963; Thornley, 1972; Iwasa and Roughgarden, 1984; Bloom et al., 1985), where plants preferentially allocate biomass to the organ responsible for the uptake of the limiting resource (Poorter et al., 2012), the manner by which the saplings in both drought treatments arrived there is very distinct. In the LD saplings, the proportional increase in root mass was the result of a differential allocation toward the root system, while the increase seen in saplings in the FD treatment was mostly the result of a differential mortality of organs. Further, the saplings exposed to the heterogeneous water availability responded with significant increases in leaf, stem and root mass, but attained the higher root mass ratio (also root to shoot ratio) by allocating more carbon to root growth relative to shoot growth. In contrast, the increase in the root mass ratio of the FD saplings was the result of terminated primary growth and a greater net tissue loss in above- vs. belowground parts; this increase would have been even larger if we had discounted the abscised leaves in this treatment. Leaf and branch shedding have been hypothesized as key drought adaptations in *Populus* species, as it decreases transpiration loss through leaf area (Rood et al., 2000; Galvez et al., 2011). The loss in root mass in the FD saplings was more difficult to discern, as the root mass of the FD saplings was similar to the initial root mass, it would appear that the root system of these saplings was maintained during the drought conditions. However, this observation is not supported by the reduction in root volume of the FD saplings from the initial volume, indicating that significant root death occurred (Table 1). It appears most likely that fine root mass was shed in these root systems (Figure 2C), while coarse roots remained viable and alive (Figure 2B). This is further supported by an increase in structural root density we observed in the FD treatment (Table 1), which might also indicate that only roots with higher densities were maintained. In grasses it had been observed that roots with higher density tend to have longer life spans than roots with lower density which are more likely to die (Ryser, 1996). Similar fine root deaths have been observed in other studies exploring drought and carbon limitation in aspen (Galvez et al., 2011, 2013; Wiley et al., 2017). Our observation of fine root and leaf abscission also supports the hypothesis that the more distal parts of plants whose role is primarily resource acquisition are potentially more expendable and/or more prone to damage under stress compared to other parts, such as larger diameter roots, stems and branches, which required significant investments over time and have additional crucial functions such as transport and storage within a plant (Kozlowski, 1973; Zimmerman, 1983; Tyree et al., 1993; Sperry and Ikeda, 1997; Landhäusser and Lieffers, 2012; Wiley et al., 2017). This may be especially important when considering a tree species (here aspen) with a root system that is adapted for long-distance resource acquisition (Snedden, 2013), as well as for the storage of NSC as reserves for post-disturbance regeneration (Wiley et al., 2019). Similar adaptations in root allocation have also been observed in other species growing in environments prone to water limitation and fire disturbance (Bell et al., 1996; Hoffman et al., 2004; Tomlinson et al., 2012).

The continued carbon acquisition in saplings under localized drought combined with an increased investment of carbon into the growth and maintenance of the root system allowed these plants to quickly adapt and generate new leaf mass and leaf area similar to the WW saplings, suggesting continued investment in leaf area development and its maintenance even under locally reduced water availability. Our results suggest that when a plant experiences drought but has access to areas in the soil that are less or non-limiting in moisture availability, the plant should be able to compensate. If the plant is able to use the portion of the root system that experiences the non-limiting conditions, it could potentially maintain the rest of the root system and continue to support aboveground growth and prevent leaf abscission. Interestingly, the LD saplings had overall similar total aboveground mass compared to the WW saplings, but they had less stem structural mass, suggesting that the WW saplings might have prioritized stem growth over other potential carbon allocation strategies. Instead, LD saplings increased stem starch concentrations from initial, which suggests a reserve storage strategy to potentially increase chances of rapid growth when conditions improve or for re-filling of xylem vessels when drought conditions worsen and cavitation occurs (Brodribb et al., 2010; Sala et al., 2012; Trifilò et al., 2017; Trugman et al., 2018). There were no differences in the overall NSC concentration of the combined root system among the three treatments. However, the FD saplings had two times higher soluble sugar concentration in the roots (9.54% dry weight) compared to the root system portion in the dry soil of the LD treatment (4.85% dry weight), which suggests a significant osmotic adjustment to improve the acquisition of water from the soil and avoid water loss back to the soil. This response is expected as it is well established that under drought stress, a higher solute concentration in roots will reduce their water potential and therefore increase the passive movement of water from the soil into the roots, in an attempt to relieve plant stress (Chaves, 1991; Gebre et al., 1998; Arndt et al., 2001; Kozlowski and Pallard, 2002; Galvez et al., 2011). Interestingly, while the localized drought conditions also led to an increase in soluble sugar concentration in the drought exposed portion of the root system, this increase was over 30% (4.85% dry weight) relative to the wet roots. This differential response of sugar concentration between the roots in the full and the localized drought might suggest a different strategy for how these plants cope with low soil water availability affecting only a portion of a root system (see below).

The fact that there was a striking difference between the degree of sugar accumulation between a full droughted root system (FD) and a partially droughted root system (LD) indicates that homo- or heterogeneous water limitations trigger different allocation responses within a root system. When faced with heterogeneous soil moisture availability, a root system showed very distinct patterns of structural and reserve mass allocation. Under the localized drought conditions, structural mass was allocated toward the portion of the root system that experienced the non-limiting condition and led to overall higher root mass and root volume. The increased allocation to root growth in the wetter soil likely allowed the saplings to compensate for the reduced water uptake in the part of the root system that experienced the low moisture availability, allowing the plant to maintain gas exchange and growth rates aboveground that were similar to those in saplings experiencing no water limitation. Additionally, the portion of the root system that experienced the limiting conditions likely preserved some of its functionality, as it maintained its mass and volume. We speculate that the preservation of the drying portion of the root system may have been favored in comparison to complete abscission, as the sapling had already invested in its establishment and the roots may be able to assist with future resource capture if the soil becomes rewetted. The benefits of maintaining a drying portion of a root system in comparison to complete abscission have been considered in other species subjected to heterogenous soil moisture conditions (Kosola and Eissenstat, 1994; Fort et al., 1997).

Since saplings in the LD treatment had access to water from the wet pot and the root system proliferated in these conditions, the demand for water supply from the dry portion of the root system was low, resulting in a reduced for need for osmotic adjustment via sugar accumulation (see above). However, the dry portion of the root system in a LD sapling accumulated significantly more starch than the wet portion of the root system, which might indicate a preferential allocation toward storage of reserves in the drier portion of the root system. These reserves would be available for future translocation or remobilization for growth, reproduction and/or other physiological processes such as osmotic adjustments in case drought conditions persist or worsen. This increase in NSC concentration in the dry portion of the root system could be driven by several possible mechanisms and processes within a plant. As mentioned previously, the lower soil water potential in the drought-exposed roots could have induced an active solute buildup for osmotic adjustment to allow for improved water uptake or the adjustment was more passive, where a lower turgor in the dry portion of the root system may have limited its growth, leading to an accumulation in NSCs due to reduced growth demand (Körner, 2003). Alternatively, the heterogeneous water availability could also have created a steeper gradient in water potentials across the entire root system, allowing for lateral water redistribution within the root system, increasing the hydration and with that the maintenance of functionality in the drought-exposed portion of the root system. Similar responses have been observed in other studies and species (Burgess and Bleby, 2006; Bleby et al., 2010; Prieto et al., 2012). By hydrating the roots experiencing water limitations from the portion of the root system that experienced less or non-limiting conditions, the risk of root cavitation and desiccation of the drought-exposed roots was likely reduced and these roots were more likely to maintain contact with the soil, enabling continued resource acquisition and other functional interactions such as mycorrhizae (Bleby et al., 2010).

This study highlights the importance of considering spatial heterogeneity of belowground resources when explaining above- and belowground responses of trees to stress. This is particularly important when studying mature trees that have extensive root systems. These trees most likely experience considerable vertical and lateral moisture gradients in the rooting space and within a root system. Since our application of the drought treatment was applied at the pot-level to assure that the drought at the root level was comparable, we recognize that the drought effects at the whole plant level were different among our treatments. Exploring these relationships and responses on these root systems in greater detail is further complicated by the generally poor accessibility of whole root systems (Hartmann et al., 2018). However, the results of our study demonstrate an adaptability and a multi-faceted response of root systems of a perennial species exposed to heterogeneous soil moisture environments. Depending on the soil moisture conditions, the root systems we studied exhibited plasticity in carbon allocation between structural mass and NSC, with differences in allocation between and within organs. The aspen saplings appeared to optimize functionality of the root system during water limiting conditions that affected only a portion of the root system by increasing root volume where water was locally available and preferentially accumulating additional NSC where root growth was limited. Our study highlights a need for exploring other potential measures of carbon allocation under stress, such as measures of total carbon and nitrogen. Short-term responses, like those noted here, will likely have impacts on how a plant will react to subsequent changes in stress conditions and might play a role in the adaptation and/or acclimation processes that have been observed in perennial plants exposed to stress over the short- and long-term (Rachmilevitch et al., 2008; Pomiès et al., 2017). In our short-term study, aspen saplings responded relatively rapidly to moisture stress by enhancing the functionality of the entire root system through adaptation [increase in root system size (LD) or leaf and root loss (FD)] and acclimation processes (accumulation of reserves), which can be considered beneficial even under prolonged drought conditions, as roots are a critical organ in aspen not only for resource uptake, but also for maintaining its resilience (i.e., vegetative regeneration) to disturbances.

## Data Availability Statement

The raw data supporting the conclusions of this article will be made available by the authors, without undue reservation.

## Author Contributions

AH, MM, and SL crafted the main research questions and experimental design. AH and MM collected the data and performed the data analyses. AH wrote the manuscript. MM, EW, and SL revised the manuscript. All authors contributed to the article and approved the submitted version.

## Conflict of Interest

The authors declare that the research was conducted in the absence of any commercial or financial relationships that could be construed as a potential conflict of interest.
