# Supplementary material for: Splitting the Difference: Heterogeneous Soil Moisture Availability Affects Aboveground and Belowground Reserve and Mass Allocation in Trembling Aspen
Source: Front Plant Sci. 2021 May 14;12:654159. doi: 10.3389/fpls.2021.654159 (PMC8160524; doi:10.3389/fpls.2021.654159)
Supplement: Supplementary file 1 [file Data_Sheet_1.docx]

Supplementary Material

# Supplementary Figures and Tables

## Supplementary Figures


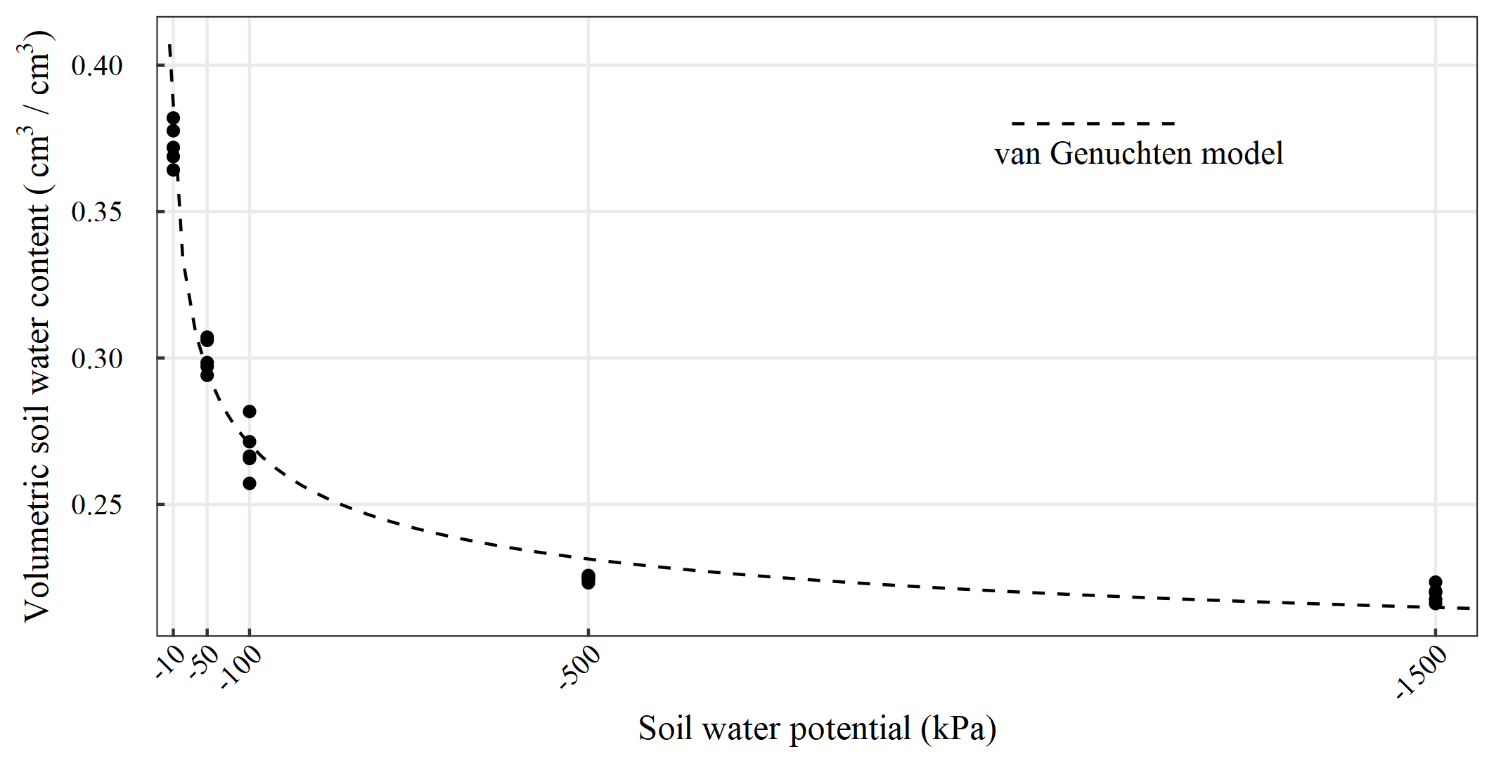


**Supplementary Figure S1.** Soil water retention curve determined using the pressure extractor method (Reynolds and Clarke Topp, 2008) for the sandy-loam soil used to fill split-pots. Soil hydraulic properties were assessed to determine use of both MPS2 sensors and daily weighing to produce moderate, progressive drought conditions. The van Genuchten model was used to represent the relationship between volumetric soil water content (SWC) and soil water potential (SWP); $SWC=\theta_{r}+\frac{\theta_{s}-\theta_{r}}{{(1+({\alpha SWP)}^{n})}^{1-1/n}}$. The estimation of the four parameters are: $\theta_{r}\sim0.18; \theta_{s}\sim0.49; \alpha\sim0.32; n\sim1.35$


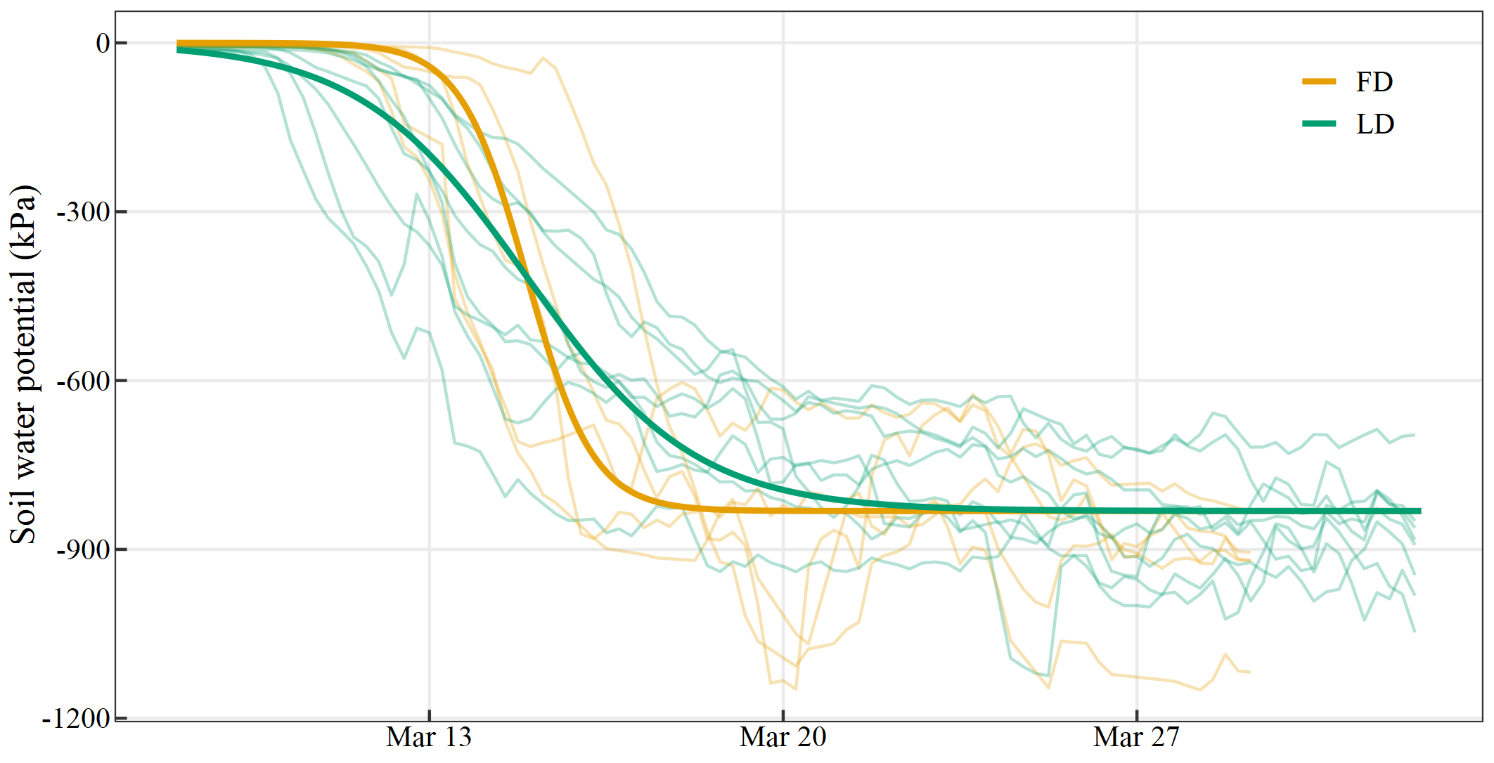


Supplementary Figure S2. Change in soil water potential (kPa) measured using MPS2 sensors for full drought (FD, yellow) and localized drought (LD, green) treatments over the four-week experimental period. A logistic function was used to represent the progressive decrease in soil water potential over time for the two soil moisture treatments: $SWP=\frac{Asym}{1+ e^{{(x_{mid}-Time)}/{scal}}}$. The estimated parameters are: $Asym\sim-831.8; x_{mid}\sim167.3; {scal}_{FD}\sim15.8; {scal}_{LD}\sim39.8$.

## Supplementary Tables

Supplementary Table S1. Schedule of the growth chamber settings (daylight length, air temperature (°C), light level (µmol) and humidity (%) between November 21^st^, 2016 and March 5^th^, 2017 when the saplings were cycled through winter and spring conditions and during the experimental period starting March 6^th^, 2017.

| **Date** | **Day length** | **Hour interval** | **Temperature** | **Light Level** | **Humidity** |
| --- | --- | --- | --- | --- | --- |
| Nov 21^st^ 2016 – Jan 3^rd^ 2017 | 0h | 0:00 – 0:00 | -1°C | 0 µmol | Turned off |
| Jan 4^th^ – Jan 19^th^ 2017 | 12 h | 7:00 – 10:00 | 5°C | 500µmol | 60% |
|  |  | 10:00 – 14:00 | 5°C | 500µmol | 60% |
|  |  | 14:00 – 19:00 | 5°C | 500µmol | 60% |
|  |  | 19:00 – 7:00 | 2°C | 0µmol | 60% |
| Jan 19^th^ – Feb 3^rd^ 2017 | 14 h | 6:00 – 10:00 | 9°C | 500µmol | 60% |
|  |  | 10:00 – 14:00 | 12°C | 500µmol | 60% |
|  |  | 14:00 – 20:00 | 9°C | 500µmol | 60% |
|  |  | 20:00 – 6:00 | 6°C | 0µmol | 60% |
| Feb 3^rd^ – Feb 6^th^ 2017 | 16 h | 5:00 – 10:00 | 13°C | 500µmol | 60% |
|  |  | 10:00 – 14:00 | 18°C | 1000µmol | 60% |
|  |  | 14:00 – 21:00 | 16°C | 1000µmol | 60% |
|  |  | 21:00 – 5:00 | 10°C | 0µmol | 60% |
| Feb 6^th^ – Feb 22^nd^ 2017 | 12 h | 8:00 – 20:00 | 18°C | 500µmol | 60% |
|  |  | 20:00 – 8:00 | 16°C | 0µmol | 60% |
| Feb 22^nd^ – April 1^st^ 2017 | 24 h | 0:00 – 0:00 | 20°C | 500µmol | 60% |
